# Detection of the Entomopathogenic Fungus *Beauveria bassiana* in the Rhizosphere of Wound-Stressed *Zea mays* Plants

**DOI:** 10.3389/fmicb.2018.01161

**Published:** 2018-06-11

**Authors:** Aimee C. McKinnon, Travis R. Glare, Hayley J. Ridgway, Artemio Mendoza-Mendoza, Andrew Holyoake, William K. Godsoe, Jennifer L. Bufford

**Affiliations:** ^1^Bio-Protection Research Centre, Lincoln University, Christchurch, New Zealand; ^2^The New Zealand Institute for Plant & Food Research Limited, Christchurch, New Zealand

**Keywords:** entomopathogenic fungi, endophytes, plant interaction, agroecosystems, biocontrol, multitrophic interactions

## Abstract

Entomopathogenic fungi from the genus *Beauveria* (Vuillemin) play an important role in controlling insect populations and have been increasingly utilized for the biological control of insect pests. Various studies have reported that *Beauveria bassiana* (Bals.), Vuill. also has the ability to colonize a broad range of plant hosts as endophytes without causing disease but while still maintaining the capacity to infect insects. *Beauveria* is often applied as an inundative spore application, but little research has considered how plant colonization may alter the ability to persist in the environment. The aim of this study was to investigate potential interactions between *B. bassiana* and *Zea mays* L. (maize) in the rhizosphere following inoculation, in order to understand the factors that may affect environmental persistence of the fungi. The hypothesis was that different isolates of *B. bassiana* have the ability to colonize maize roots and/or rhizosphere soil, resulting in effects to the plant microbiome. To test this hypothesis, a two-step nested PCR protocol was developed to find and amplify *Beauveria in planta* or in soil; based on the translation elongation factor 1-alpha (*ef1α*) gene. The nested protocol was also designed to enable *Beauveria* species differentiation by sequence analysis. The impact of three selected *B. bassiana* isolates applied topically to roots on the rhizosphere soil community structure and function were consequently assessed using denaturing gradient gel electrophoresis (DGGE) and MicroResp^TM^ techniques. The microbial community structure and function were not significantly affected by the presence of the isolates, however, retention of the inocula in the rhizosphere at 30 days after inoculation was enhanced when plants were subjected to intensive wounding of foliage to crudely simulate herbivory. The plant defense response likely changed under wound stress resulting in the apparent recruitment of *Beauveria* in the rhizosphere, which may be an indirect defensive strategy against herbivory and/or the result of induced systemic susceptibility in maize enabling plant colonization.

## Introduction

Isolates of *Beauveria bassiana* (Balsamo) Vuillemin *sensu stricto* exhibit considerable variation in pathogenicity toward insects. Typically, the criteria for selection of isolates for biocontrol purposes are based on observed insect mortality rates in bioassays, in addition to the efficiency of producing conidia in culture. With the primary focus on pathogenicity, suitability for the environmental conditions and ability of a *B. bassiana* isolate to continue to function within the environment intended for application have often not been considered (Meyling and Eilenberg, 2007). With evidence mounting of *B. bassiana* having an opportunistic endophytic strategy to its lifecycle (Vidal and Jaber, 2015; McKinnon et al., 2017), it is worthwhile to consider whether there is variation within *B. bassiana* species for the ability to colonize plant tissue (Kia et al., 2017). For example, in soil it is unknown whether certain isolates occupy only specific tissues or organs of plants, such as on the rhizoplane, rather than the internal tissues of roots. Recent histological studies indicate that internal (endophytic) colonization of leaf tissues, in particular, is either an uncommon phenomenon, is transient in nature or is not possible without damaged tissue for the entomopathogenic fungi to enter (Landa et al., 2013; Ullrich et al., 2017; Koch et al., 2018). Further studies are yet required to understand whether there is isolate variation in plant colonization ability of leaf and root tissues.

Generally, the below-ground functions of the plant microbiome are predominantly nutritional, with various and ubiquitous mycorrhizal and rhizobial associations that enhance the access of the host plant to organic and inorganic soil nutrient reserves (Bacon and White, 2016). Entomopathogenic fungi from the genus *Metarhizium* are considered common rhizosphere colonizers in many ecosystems. These fungi are also pathogenic to various insect species, the cadavers of which may provide a source of available nitrogen to plants (Bruck, 2010; Behie et al., 2013). Behie et al. (2012) demonstrated that the root colonizing capability and insect pathogenicity of *Metarhizium* can be coupled to translocate nitrogen to plant roots via the fungal mycelial network from an insect cadaver. Species of *Beauveria* are also frequently isolated from soil (Jaronski, 2008), therefore, *B. bassiana* isolates that have the capacity to both interact with plant tissues below-ground and infect insects may also transfer nitrogen from insect cadavers to plants (Barelli et al., 2016), although this has not been demonstrated.

In exchange for nitrogen, endophytic and rhizosphere competent species of *Metarhizium* or *Beauveria* could acquire plant-derived carbon, such as raffinose (Fang and St. Leger, 2010) or sucrose, as has been demonstrated for root-endophytic isolates of *Trichoderma virens* (Vargas et al., 2009). In nature, the resident soil microbial community may interact with inocula to affect carbon utilization through competition and consequently also inhibit the colonization of plant roots or persistence after initial establishment (Lugtenberg et al., 2002). On the other hand, inundative inoculation of these fungal agents into the plant rhizosphere may impact the resident microbial community by altering the composition or function. The European Union has consequently included, as part of the criteria in the registration process of new biological pesticides, an assessment of potential effects to indigenous soil microorganisms arising from the addition of biological control agents (Commission regulation No. 544/2011). However, there is little research available about the potential effects of applying entomopathogenic fungi to soil in particular (Hu and St. Leger, 2002; Hirsch et al., 2013; Mayerhofer et al., 2017).

Soil microbial communities are extremely diverse in composition and function, and are involved in many processes such as organic matter decomposition and mineralization, nutrient mobilization and carbon sequestration (Reynolds et al., 2003). Consequently, the loss of microbial functional diversity in soil is effectively an indicator for decrease in soil quality (Chapman et al., 2007). Community-level physiological profiles (CLPPs) are assessed by carbon substrate utilization and enable the evaluation of the microbial functional diversity of a given soil. CLPPs have been accomplished using methods such as Biolog^TM^ and MicroResp^TM^ (Campbell et al., 2003; Calbrix et al., 2005). These methods therefore provide an excellent tool to assess microbial diversity and soil functioning following the application of inocula, like *B. bassiana*, to the rhizosphere microbiome.

Rhizosphere dwelling microorganisms can prime the plant defensive response to either inhibit or enhance pathogenic/endophytic invasion and herbivore attack through the induction of certain plant resistance gene pathways (Pieterse et al., 2012). For example, a recent study by Haney et al. (2017) demonstrated that certain rhizosphere dwelling strains of *Pseudomonas* induce systemic susceptibility (ISS) to foliar pathogens in *Arabidopsis*, yet they simulaneously enhance resistance to the chewing insect *Trichoplusia ni* (cabbage looper) via activation of the plant jasmonic acid (JA) pathway. In their study, ISS was found to result from the suppression of the salicylic acid (SA) defense pathway associated with pathogen resistance, suggesting a kind of signaling trade-off between JA and SA when under attack (Pieterse et al., 2012). Inversely, when plants are subjected to stress such as that resulting from insect herbivory or wounding, they elicit a suite of defensive compounds in response (Howe and Jander, 2008). In the rhizosphere, changes in root exudates resulting from this plant defensive response can impact the microbial community diversity, density and activity to shape the functional microbiome (Pangesti et al., 2013). Thus the complexity of the microbiome and the pleitropic effects in the plant host resulting from these various interactions may be important to consider, both to understand the life history strategy of the fungi and in order to determine the potential outcome of introducing *Beauveria* for biocontrol purposes.

The primary aim of this study was to investigate the interaction of *B. bassiana* in the rhizosphere of maize (*Zea mays*), by assessing for differential effects on the resident microbial community as a result of inoculation with three *B. bassiana* isolates. Potential changes in the microbiome of the rhizosphere were investigated, by inducing plant host stress through intensive wounding of the foliage. We hypothesized that survival in the rhizosphere of the *Beauveria* inoculum may be influenced by plants recovering from intensive foliar damage. In order to monitor our *Beauveria* isolates in the rhizosphere, a novel nested PCR protocol was first developed with primers designed from the translation elongation factor 1-alpha gene (*ef1α*) to enable the detection of *Beauveria* from plant material and rhizosphere soil, while excluding non-target fungi and the plant-host DNA (maize). In previous literature, a method for the molecular detection of endophytic *B. bassiana* from plant tissues was reported (Landa et al., 2013; Quesada-Moraga et al., 2014; Tall and Meyling, 2018). This method adopted the use of two primer sets from the internal transcribed spacer (ITS) region of ribosomal DNA (rDNA), for a two-step nested protocol. However, these primers were designed to amplify a specific isolate of *B. bassiana*, isolate EABb 04/01-Tip and not to amplify ITS1-5.8S-ITS2 sequences of opium poppy *Papaver somniferum* or other fungi different from *B. bassiana* (Landa et al., 2013).

The PCR protocol herein was initially developed to allow rapid and sensitive screening of plant material and soil in order to potentially test for multiple species of *Beauveria* (see thesis by McKinnon, 2017). The primers designed were therefore intended to be less specific than those previously reported by Landa et al. (2013), in order to potentially test and/or detect other *Beauveria* species that may be present apart from the inocula.

## Materials and Methods

### Fungal Cultures and Inocula Preparation

Three *B. bassiana* isolates (BG11, FRh2 and J18) were used in this study (**Table 1**). BG11 was isolated from wild dandelion root *Bellis perennis* (Asterales: Asteraceae), FRh2 from the cadaver of a pine bark beetle (*Hylastes ater*) and J18 from maize leaf, isolated during a natural endophyte survey of maize (Brookes, 2017).

**Table 1:** Table of fungal isolates used in this study.

Isolate	Accession/ Depository^1^	Species	Host (insect/plant)^2^	District, New Zealand	Isolated
BG11	BMCC, BPRC	*Beauveria bassiana*	*Bellis perennis (Asterales: Asteraceae)* root	Christchurch	A. Clousten
E1063	MH165265	*B. bassiana*	*Vespula germanica* (Hymenoptera: Vespidae)	Nelson	N. Cummings
E1069	MH165266	*B. bassiana*	*V. germanica*	Nelson	N. Cummings
FBHU	MH165264	*B. bassiana*		Selwyn	A. McKinnon
FRh2	MH165263	*B. bassiana*	*Hylastes/Hyalurgis*	Auckland	S. Reay
E1079	MH165253	*B. caledonica*	*Dermaptera*	Nelson	N. Cummings
FRh1	MH165255	*B. caledonica*	*Hylastes/Hyalurgis*	Auckland	S. Reay
NC142	MH165256	*B. caledonica*	*Prionoplu reticularis* (Coleoptera: Cerambycidae)	Westland	N. Cummings
NC44	MH165252	*B. caledonica*	n.d.	Taupo	N. Cummings
NC49	MH165254	*B. caledonica*	Coleoptera	Taupo	N. Cummings
E1059	MH165247	*B. malawiensis*	*Vespula vulgaris* (Hymenoptera: Vespidae)	Westland	N. Cummings
E1060	MH165250	*B. malawiensis*	*V. vulgaris*	Westland	N. Cummings
NC215	MH165248	*B. malawiensis*	*V. vulgaris*	Westland	N. Cummings
NC220	MH165249	*B. malawiensis*	Orthoptera: Tettigoniidae	Westland	N. Cummings
NC222	MH165251	*B. malawiensis*	*Vespula* sp.	Westland	N. Cummings
E1067	MH165262	*B. pseudobassiana*	*Vespula* sp.	Nelson	N. Cummings
E1080	MH165260	*B. pseudobassiana*	Coleoptera: Scarabaeidae	Nelson	N. Cummings
E1083	MH165257	*B. pseudobassiana*	n.d.	Nelson	N. Cummings
E1139	MH165258	*B. pseudobassiana*	*Phaulacridium marginale (Orthoptera: Acrididae)*	Nelson	N. Cummings
E1175	MH165261	*B. pseudobassiana*	*V. vulgaris*	Nelson	N. Cummings
NC209	MH165259	*B. pseudobassiana*	*Costelytra zealandica* (Coleoptera: Scarabaeidae)	Westland	N. Cummings
J1	MH165245	*Alternaria alternata*	*Pinus radiata* needle	Canterbury	J. Brookes
J8	MH165246	*Fusarium oxysporum*	*Pinus radiata* needle	Canterbury	J. Brookes
J10	MH165244	*Aspergillus nidulans*	*Pinus radiata* needle	Canterbury	J. Brookes
J18	MH165267	*B. bassiana*	*Zea mays* L. leaf	Canterbury	J. Brookes
LU132	BMCC, BPRC	*Trichoderma atroviride*			
C14	BMCC, BPRC	*Metarhizium anisopliae*	n.d.	Canterbury	M.C. Lefort
ICMP-1	ICMP-11019	*Lecanicillium lecanii*	*Cecidophyopsis ribis* Westwood (mite)	Timaru	W Thomas
ICMP-2	ICMP-14476	*Verticillium dahliae*	*Vitis vinifera* L. (Rhamnales: Vitaceae)	Marlborough	M. Braithwaite

*^*1*^Accession numbers are based on nucleotide sequence data of ITS1 rRNA or *ef-1α* submitted to the National Center for Biotechnology Information (NCBI) database. Depository numbers are alternatively provided where molecular identities are unavailable, for either the International Collection of Microorganisms from Plants (ICMP) or the Biocontrol Microbial Culture Collection (BMCC), Bio-Protection Research Centre (BPRC), Lincoln University, New Zealand. ^*2*^n.d., not determined, absent information indicates soil or other source for isolation.*

Inocula were prepared as conidial suspensions for direct application to roots of maize seedlings. Suspensions were produced from cultures grown on potato dextrose agar (PDA; Difco, BD, United States) after 3 weeks at 20°C. Five mL of sterile 0.05% (v/v) Tween 80 was added to each plate (with five plates per isolate), scraped gently with a hockey stick to blend conidia and then poured through two layers of Miracloth^TM^ (Merck Millipore) to obtain 25 mL of conidial suspension per isolate. The concentration of conidia per mL was calculated based on counts made from 10 μL of a 10^-2^ dilution placed on a Neubauer hemocytometer counting chamber. Conidial concentrations were then adjusted in 0.05% Tween 80 based on the hemocytometer calculation to achieve 10^7^ conidia per mL in 180 mL volumes per isolate, for immediate application to maize seedlings by root dip.

To check the viability of conidia for each isolate, 100 μL of a 10^-5^ dilution from each suspension was spread onto PDA, with three replicates per suspension and incubated for 10 days at 20°C. After 10 days, the number of colony forming units (CFUs) were counted. The average number of viable conidia per isolate was multiplied by 10 to get CFU/mL, and then the CFU/mL values were multiplied again by 10^5^ to estimate the quantity of viable conidia per mL of original suspension. By this method, the percentage of viable conidia for each isolate suspension was above 94%.

### Maize Growth and Inoculation

Maize (*Zea mays*) were grown from seed of cultivar Pioneer 34H31. Seeds were first surface sterilized by soaking them in a 2.5% sodium hypochlorite (NaClO) and 0.02% Tween 80 solution for 7 min, followed by two washes in sdH_2_O with 1 min per wash. Surface sterilization efficacy was assessed by aseptically rolling a subset of 10 surface sterilized seeds onto 10% potato dextrose agar (PDA), and then these ‘imprinted’ control plates were incubated for 14 days at 25°C to check for growing cultures (Schulz et al., 1998). Seed were placed in pairs on 1% agar in deep Petri dishes (25 × 100 mm), for incubation at 25°C for 7 days in the dark. On day five, the seedlings were inoculated by soaking the roots for 3 min each in the respective conidial suspensions. Following inoculation, the plants were immediately returned to fresh 1% agar plates (one plate per seedling) for further incubation at 25°C for 3 days. Seedlings were then planted individually in 2.5 L pots containing non-sterile pasture soil blended with river sand (4:1 silt:river sand). The soil nutrient availability was analyzed and found to be generally low, with N at 65 kg/ha N, P at 10 mg/L and K at 0.19 me/100 g (Hill Laboratories Limited, New Zealand).

Plants were subsequently maintained in a plant growth chamber for 30 days according to the following conditions: 16 h light at 25°C, 8 h dark at 20°C with a constant 68% (±2%) relative humidity. The plants were provided with a low water input regime to facilitate water deficit. Daily watering was done manually using a hose on the misting setting to provide approximately 4 mm water per pot per day within the first 13 days of growth, and then watering was increased to twice daily and 5 mm each time, as the plant shoots exceeded 200 mm.

### Experimental Design

The rhizosphere experiment was arranged in a split-plot design consisting of six blocks. Within each block, four time treatments were represented as days after inoculation (DAI) in randomized order. The time treatments constituted the main-plots which were arranged further in a randomized complete block design (RCBD) by isolate treatment (sub-plots). The main-plot time treatments were 6, 15, 30, and 30 DAI with a foliage wounding treatment; designated 30+W. The isolate sub-plot treatments were the BG11, FRh2, J18 treated plants in addition to a no-inoculum control which consisted of six replicates per main-plot.

Although harvested at 30 DAI, wounding of leaves was conducted at 23 DAI to the 30+W plants, which were treated by removing approximately 33% of the leaves per plant roughly with scissors to stimulate wound stress, as a crude proxy for herbivory. Of the leaves that were cut, approximately three quarters of leaf area was removed.

### Soil Sampling

At each sample time (6, 15, 30 DAI), the total rhizosphere soil was collected from each plant. Plants were first carefully extracted from their pots and gently shaken to remove excess and/or loose soil. The roots were then gently brushed with a sterile paintbrush to remove the rhizosphere soil onto sterilized trays. Rhizosphere soil was then mixed aseptically by hand within the trays. From the collected soil of each plant, subsamples were taken and these included a 2 mL volume sample for DNA extraction and a 100 g sample at 30 DAI only for MicroResp^TM^. The remaining pot soil was used to measure soil moisture content (SMC). Five separate root fragments (5 cm in length) were taken from each plant at 10 cm below the soil line. The root fragments were washed gently in 0.05% Tween 80 and trimmed further to 1 cm pieces with a sterile blade. All soil and plant material was weighed prior to DNA isolation and the weights were later adjusted/recorded according to DNA kit protocol (see DNA Isolation). Soil for DNA extraction and MicroResp^TM^ was stored at -20°C and all root material was stored at 4°C until required for processing.

### DNA Isolation

Genomic DNA (gDNA) was first extracted from pure fungal culture of each isolate. Fungi were cultured on sterile cellophane, placed over 10% PDA (Difco, BD, United States), and incubated for 6 days at 25°C, prior to DNA extraction. Cellophane was aseptically scraped using a surgical blade to obtain pure hyphal tissue. Fungal and plant DNA was extracted using the DNeasy PowerPlant Pro Kit (Qiagen), according to the kit instructions but with the following modification: tissue lysis was conducted with the FastPrep-24^TM^ (MP Biomedicals) at 5 m/s for 30 s for fungal tissue and 5 m/s for 40 s.

Plant gDNA was obtained from 6 day old seedlings for PCR experimental optimization, and from root material and rhizosphere soil at 6, 15, and 30 DAI. Root DNA yields ranged from 18 to 22 ng/μL when quantified using a Qubit^TM^ 3.0 Fluorimeter ‘high sensitivity double-stranded DNA’ assay (Invitrogen, Thermofisher Scientific). DNA from rhizosphere soil was extracted using the DNeasy PowerSoil Kit (Qiagen) according to the protocol provided but with the following modification: lysis was conducted also with the FastPrep-24^TM^ at 5 m/s for 40 s. Qubit^TM^ high sensitivity double-stranded DNA assays were performed to assess DNA concentration and soil DNA yields typically ranged from 2 to 5 ng/μl.

### Primer Design for Selective PCR

Using the Basic Local Alignment Search Tool (BLAST) (Altschul et al., 1990) against the nucleotide database of the National Center for Biotechnology Information (NCBI), seven partial sequences of the translation elongation factor 1-alpha gene (*ef1α*) were obtained *in silico* using and including *B. bassiana* (GenBank accession: AY531924.1) as a reference query. These included *B. brongniartii* (HQ880991.1*), B. caledonica* (HQ881012.1), *Trichoderma reesei* (Z23012.1), *Verticillium dahliae* (AY489632.1), *Metarhizium anisopliae* (DQ463996.2), and *Aspergillus nidulans* (XM656730.1). Four additional *ef1α* sequences were acquired *in silico* by using the *B. bassiana* reference sequence as a query against genome sequences of *B. bassiana* (isolates K4 and E17-P), *B. caledonica* (FRh1), and *B. malawiensis* (Bweta) (unpublished, BPRC, Lincoln University) in a Stand Alone BLAST search (Zhang et al., 2000). Nucleotide alignment by ClustalW (Higgins and Sharp, 1988) was performed in Genious Pro ver. 5.6.5. (Biomatters^LTD^), resulting in a 989 bp-long nucleotide multiple sequence alignment (MSA) after trimming that was subsequently analyzed with the software package SpideR (SPecies Identity and Evolution in R) (Brown et al., 2012). Using SpideR, the *ef1α* of *Beauveria* species represented in the MSA were designated collectively as a single species vector, in order to compare the genus ‘*Beauveria*’ against the other fungal genera represented. This enabled SpideR to find possible sites of genetic variation that were unique to the ‘*Beauveria*’ vector (and thus genus) in the MSA on the *ef1α* fragment, compared to the selected outgroup.

Using the informative sites identified by SpideR, two pairs of primers were designed manually by visualization of sequences in Geneious^®^ Pro 5.6.5 for nested PCR (**Table 2**).

**Table 2:** Novel primer pairs designed for a 2-step nested PCR protocol.

Primer ID	Sequence (5′ → 3′)	TM	Amplicon (bp)	PCR type
EF3F	ACGGTGCCCGTCGGT	60	406	*Beauveria* multi-species nested pair 1
EF5R	ACTTGATGAACTTGGGGTTGTTC	55		
EF4F	GTCGCTGGTGACTCCAAGAA	59	176	*Beauveria* nested pair 2^1^, and/or qPCR
EF4R	GTACGGCGGTCGATCTTCTC	60		

*Designed from the translation elongation factor alpha-1 (*ef1α*) gene to target multiple *Beauveria* species *in planta.* Primer melting temperature (TM), the target amplicon size in base-pair (bp), and the type of experiment are described. ^*1*^Primers’ EF4F and EF4R are not specific for *Beauveria*, unless used in a two-step nested experiment.*

The first primer pair EF3F and EF5R (designated ‘EF3-5’ collectively) were designed to amplify a 406 base-pair (BP) fragment from multiple species of the *Beauveria* genus. An *in silico* test of primer specificity was conducted by using the EF3-5 primer sequences as queries in BLASTN 2.2.27 (Altschul et al., 1997) against the non-redundant GenBank database, set with parameters for the identification of short, near matches. A second pair, EF4F and EF4R (designated ‘EF4-4’ collectively) were designed to be general based on the MSA but nested within the EF3-5 amplicon and suitable for a two-step nested PCR protocol in addition to real time qPCR; i.e., the target amplicon for EF4-4 was designed to be shorter (<200 bp) to maximize amplification efficiency. Additionally, the primer sets were designed such that single nucleotide polymorphisms (SNPs) within the target amplicons generated by both primer sets enabled species differentiation for *Beauveria* and for non-target genera via sequencing analysis. The Primer Express software (Applied Biosystems, Roche, Branchburg, NJ, United States) was used as an additional *in silico* test to assess the secondary structure, dimerization, and melting temperature of the primer sets (**Table 2**). All primers used in this study were synthesized by Integrated DNA Technologies (IDT^TM^, San Diego, CA, United States).

### PCR Protocol

All standard PCR amplifications were conducted in a Kyratec SuperCycler SC300 thermal cycler. PCR reagents were prepared for 25 μl volume reactions consisting of: 1.5U/reaction Fast Start Taq DNA polymerase, 1 × buffer, 2 mM MgCl_2_, 0.2 mM deoxynucelotide triphosphate (dNTP) (Roche, Roche Custom Biotech, Switzerland), 0.2 × bovine serum albumin (BSA, Sigma-Aldrich), 0.4 mM of each primer and 2 μL of eluted DNA. The quality and size of the PCR products were assessed by agarose gel electrophoresis, using a 1% gel in 1× TAE (40 mM Tris-OH, 20 mM Acetic Acid, pH 7.8, 1 mM EDTA). No template controls (NTC) consisting of reagent master mix and ddH_2_O were including in each amplification experiment. From each amplification, 5 μL of each PCR product was loaded in the agarose gel containing a DNA gel stain (0.5x RedSafe^TM^), together alongside 7 μL of a 1kb DNA ladder (Hyperladder II, Bioline, United States). PCR products were separated by electrophoresis in 1× TAE buffer at 100 V for 30 min and then visualized following exposure to UV light using the Versadoc Imaging Systems Model 3000 (Bio-Rad, United States). All reactions were repeated at least twice and included positive controls including gDNA of *B. bassiana* and *Zea mays.* The two step nested PCR protocol was optimized to the following conditions: for standard PCR with primers EF3F and EF5R (nested PCR 1) cycling conditions were (1) 95°C for 5 min, then (2) 25 cycles consisting of: 95°C for 30 s, 65°C for 30 s, 72°C for 1 min; (3) 72°C for 7 min. Standard PCR with primers EF4F and EF4R (nested PCR 2) were (1) 95°C for 5 min, (2) 30 cycles consisting of: 95°C for 30 s, 65°C for 30 s, 72°C for 1 min; and then (3) 72°C for 7 min.

All real-time PCR amplifications used primers EF4F and EF4R (nested step 2 alternative to standard PCR) and were conducted in an Applied Biosystems StepOnePlus^TM^ Real Time PCR System (Applied Biosystems^®^). The real time PCR reagents were prepared for 16 μl volume reactions consisting of: 1 × buffer, 4 mM MgCl_2_, 0.6 mM dNTP (Roche, Roche Custom Biotech, Switzerland), 0.5 mM of each primer, 0.625 μm ROX passive reference (Invitrogen^TM^, United States), 1:30000 dilution SYBR^TM^ Green 1 (Bio-Rad Laboratories Inc, Hercules, CA, United States) and 10 ng of eluted DNA template. Cycling conditions were (1) 95°C for 2 min, (2) 30 cycles consisting of: 95°C for 15 s, 64°C for 30 s, and 72°C for 45 s; followed with an optional melting curve (3), step and hold: 95°C for 15 s, 60°C for 1 min and 95°C for 15 s.

### PCR Protocol Analysis

The nested PCR protocol was optimized by constructing a series of standard curves using real time PCR. Initially, PCR amplification efficiency was estimated for the first set of primers EF3F and EF5R (‘EF3-5’) by constructing a standard curve generated from amplification of a gDNA dilution series consisting of *B. bassiana* isolate BG11 template in ddH_2_O, which was prepared with the following concentrations: 100 ng (10^-1^), 10 ng (10^-2^), 1 ng (10^-3^), 0.1 ng (10^-4^) and 0.01 ng (10^-5^) of template gDNA, respectively.

### PCR Sensitivity and Specificity

PCR sensitivity of the two-step nested protocol was then tested by comparing two real time PCR standard curve experiments according to the protocol previously described, constructed from amplifications of: (1) EF4F and EF4R (‘EF4-4’) target amplification of diluted PCR template (1:1000), generated prior using step one primers’ EF3F and EF5R amplification of gDNA of *B. bassiana* gDNA [3.2 ng/μL]; versus (2) EF4-4 target on *B. bassiana* gDNA diluted in series, in ddH_2_O directly.

Then an additional 10-fold DNA dilution series was prepared using gDNA stock of *B. bassiana* isolate J18 [3.2 ng/μL] spiked into maize DNA [1.6 ng/μL] with ddH_2_O diluent, for a final standard curve to assess amplification sensitivity from a mixed DNA sample using the nested protocol. Template from this dilution series was subsequently included in each experimental real time PCR amplification. Mean real time PCR cycle threshold values generated from three replicated experiments that amplified the PCR template from mixed DNA were compared for consistency by statistically contrasting the mean cycle threshold values obtained using an ANOVA in R (R Stats-package) to determine PCR replication reproducibility.

Specificity of the two-step nested protocol was experimentally tested in a real-time PCR amplification experiment of a subset of fungal isolates; the DNA of which was obtained from species isolates of *B. malawiensis, B. pseudobassiana, B. caledonica, Alternaria alternata, Aspergillus nidulans, Fusarium oxysporum, Lecanicillium lecanii, Trichoderma atroviride*, and *Verticillium dahliae* (**Table 1**), in addition to maize and onion DNA (*Allium* sp.) (McKinnon, 2017); and compared against DNA template generated from *B. bassiana* isolates BG11, FRh2 and J18 used in this study.

### PCR Detection of *Beauveria* in Soil and Root Samples

PCR conducted on soil DNA was performed as previously described with the *ef1α* nested primers (EF3-5, EF4-4), however, cycle lengths were optimized to 30 cycles (step one, EF3-5) and 23 cycles (step two, EF4-4) to minimize potential non-target amplification (based on the PCR protocol analysis). PCR detection performed on root DNA samples were also optimized for the number of cycles, with 30 cycles on both steps for 6 DAI samples and 35 cycles on step 2 for the remaining samples (15, 30 DAI). Real time PCR was conducted using EF3-5 template generated from root DNA samples only, for detection of the isolates in/on roots. Amplifications were conducted from 1/1000 dilutions of PCR product using the EF4-4 primers, to ascertain PCR detection cycle thresholds for comparison of the isolate treatments.

The presence or absence of *B. bassiana* was compared independently in roots and in soil for the three isolates (BG11, FRh2, J18) relative to the control over time (6, 15, 30 DAI), by calculating the percentage present in samples. All positive bands were sequenced to confirm identify, and non-targets were subsequently excluded from the data analysis. Statistical analysis of the 30 DAI soil data was conducted using a generalized linear model (GLM) in R (v. 3.2.3, package stats 3.2.2), based on the binomial error distribution; with two factors to explain variance including ‘isolate’ and ‘wounding’ relative to the experimental blocking structure. The model was visualized with coefplot (v. 1.2.4) and assessed for fit using a chi-squared analysis of deviance test. Treatment means were contrasted using Tukey contrasts in a general linear hypothesis (GLH) multiple comparisons procedure (package multcomp 1.4-5).

### Microbial Community Composition Analysis

Rhizosphere DNA from 30 DAI samples were amplified in PCR experiments to assess whether the inoculum treatments and/or the wounding treatment had any effect on the soil microbial community composition. Using PCR protocol(s) optimized for denaturing gradient gel electrophoresis (DGGE), four target groups were successfully amplified for each soil treatment: Alphaproteobacteria, Betaproteobacteria, general fungi and arbuscular mycorrhizal fungi (AMF). Briefly, the V3 hypervariable region of the bacterial 16S rRNA gene was amplified by PCR for Alphaproteobacteria (primers’ F203α-L1401 and 341FGC-518R) and Betaproteobacteria (Beta359F-Beta682R and 518FGC-Beta682R) using nested PCR protocols previously described (Gomes et al., 2001; Muhling et al., 2008); except with a modification of 30 cycles in the first PCR for the Alphaproteobacteria group (Wicaksono et al., 2016) (**Table 3**). The small subunit (18 s) of rRNA was amplified for general fungi (primers’ AU2-AU4 and FF390-FR1GC) (Muyzer et al., 1993; Vainio and Hantula, 2000) and for the AMF group (AML1-AML2 and Glo1-NS31GC) (Simon et al., 1992; Lee et al., 2008) according to protocols previously described (**Table 3**). Reagents for all groups consisted of 25 μL volume reactions containing 1x buffer, 0.2 mM dNTPs, 1.5 mM MgCl_2_, 0.4 μM of each forward and reverse primers (IDT, Integrated DNA Technologies Inc) and 1 U *Taq* DNA polymerase (Roche, Roche Custom Biotech, Switzerland).

**Table 3:** PCR primers and thermal cycling conditions used for each target group in preparation for denaturing gradient gel electrophoresis (DGGE).

Group	Primer sets	Thermal cycling conditions
Alphaproteobacteria	F203A and L1401 341GC and 518R	96°C 4 min, 30 × (94°C 1 min, 64°C 2 min, 74°C 1 min), 74°C 10 min. 96°C 4 min, 30 × (96°C 1 min, 56°C 30 s, 74°C 1 min), 74°C 10 min.
Betaproteobacteria	β359F and β682R 518FGC and β682R	96°C 4 min, 30 × (94°C 1 min, 63°C 1 min, 74°C 1 min), 74°C 10 min. 96°C 4 min, 30 × (96°C 1 min, 60°C 1 min, 74°C 1 min), 74°C 10 min.
Fungi	AU2 and AU4 FF-390 and FR1-GC	95°C 3 min, 35 × (94°C 1 min, 50°C 1 min, 72°C 1 min), 72°C 7 min. 95°C 2 min, 8 x (95°C 30 s, 55-48°C1 30 s, 72°C 1 min), 27 × (95°C 30 s, 47°C 30 s, 72°C 1 min) 72°C 7.5 min.
Arbuscular mycorrhizal fungi	AML1 and AML2 Glo-1 and NS 31-GC	95°C 3 min, 35 × (94°C 1 min, 50°C 1 min, 72°C 1 min), 72°C 7 min. 95°C 3 min, 35 × (94°C 45 s, 52°C 45 s, 72°C 1 min), 72°C 7 min.

Denaturing gradient gel electrophoresis was performed in a Cipher DGGE Electrophoresis System (CBS Scientific). Ten milliliter of each PCR product was loaded along with 10 μL loading dye into an 8% (w/v) polyacrylamide gel (acrylamide/bis solution, 37.5:1) containing a linear denaturing gradient of 40–60% for Alphaproteobacteria; 40–55% for Betaproteobacteria; 25–55% for the general fungi and 30–45% for AMF. The 100% denaturant consisted of 7 M urea and 40% (v/v) formamide. The gels were run in 0.5 × TAE buffer for 18 h at 60 V and 60°C for Alphaproteobacteria, Betaproteobacteria; and for 17 h at 90 V for general fungi and AMF. To calibrate gels, a single sample (soil DNA sample from a BG11 treatment pot#7) was added to the first lane of every gel as a reference marker. The gels were stained with 0.1% (wt/vol) silver nitrate. Gels were developed with a sodium hydroxide and formaldehyde solution [0.01% (v/v)]. Gels were washed in a fixative solution (40% ethanol, 2% acetic acid in water) and then Cairns’ preservation solution (25% ethanol, 10% glycerol in water) for subsequent gel drying.

Analysis of the microbial community profiles was performed in Phoretix 1D Pro Gel Analysis (TotalLab, United Kingdom). The presence/absence of bands identified in Phoretix were exported as binary data to R for statistical analysis using the Vegan Package (v 2.3-5). Resemblance matrices for community profiles were built by calculating similarities between each pair of samples using the Jaccard coefficient. To visualize information on these pairwise similarities, we used non-metric multidimensional scaling (nMDS) ordination plots which were generated using ggplot2 (v. 2.1.0) to interpret multivariate distance between samples by treatment factors (isolate and wounding).

On each gel lane, one band was considered as one taxonomic unit. The number of bands per lane was then summed and used as an indicator of species richness. Species richness was analyzed first with Adonis (permutational multivariate analysis of variance using distance matrices; Vegan) performed on a distance matrix produced using the Bray-Curtis similarity coefficient, and then also by individual the groups (i.e., total fungi, AMF, Alphaproteobacteria, Betaproteobacteria) using univariate GLM in R, based on the Poisson error distribution; with two factors to explain variance including ‘isolate’ and ‘wounding’ relative to the experimental blocking structure to determine the significance.

### Microbial Community Functional Diversity Analysis

Functional diversity of soil communities was analyzed using the MicroResp^TM^ experimental method described by Campbell et al. (2003). This technique combines the advantages of the Biolog^TM^ microplate system, with those of the substrate-induced respiration (SIR) approach (Degens and Harris, 1997), providing the ability to measure CO_2_ production following short-term incubation of a whole soil microbial community as an indicator of functional diversity. Soil samples are added to the MicroResp^TM^ plate (ThermoFisher, New Zealand) along with assorted carbon substrates. Prior to conducting the assay, the soil samples required adjustment of the gravimetric soil water content (GWC) to 40% of the water-holding capacity, so that with the addition of the carbon substrate solution, the final GWC of the soil was 60% of its water-holding capacity. GWC was measured as the difference between fresh and oven-dried (120°C for 24 h) samples. A pooled and mixed sample obtained from the rhizosphere of multiple experimental plants was used to enable calculation of the GWC, by weighing the sample before and after drying it in the oven at 120°C overnight.

To prepare the soil for the MicroResp^TM^ assay, 100 g of soil was collected from the upper rhizosphere (5 -200 mm depth) of all the 30 DAI maize plants and stored at 4°C. The soil was processed through a 2 mm sieve to eliminate large aggregates, stones and roots. Approximately 0.45 g of fresh soil was added per well, per plate, and then each plate was sealed in a sealable plastic bag for incubation at 20°C for 7 days. Prior to adding the different carbon substrates, the plate containing the indicator gel was read with an absorbance microplate reader at 570 nm (spectrophotometer). The carbon substrates were then added to the 1.2 mL wells containing the rhizosphere samples at a concentration of 20 mg g-1 dry soil (calculated using the GWC obtained for each sample) per substrate. The substrates used in this experiment were: L-arabinose, D-fructose, D-galactose, α-D-glucose, D-Xylose, maltose, sucrose, raffinose, citric acid, glycoloc, tartaric, glycerol 50%, D-(+)-glucosamine hydrochloride, urea, triton x-100, L-proline, glycine, L-alanine, arginine, L-serine, cysteine, and tryrosine. Two water only substrate controls were included in the experiment. The carbon substrates used were considered representative of what may be present in plant root exudates. The MicroResp plates were then sealed and incubated for 4 h at 20°C. Following incubation, the plates were read again at 570 nm.

MicroResp^TM^ data was analyzed within the software Primer-7 (Clarke and Gorley, 2015) and also in R (v. 3.2.3) for further analyses (described below). In Primer-7, all data was first normalized and a distance matrix produced using the Euclidean coefficient. The ‘isolate’ and ‘wounded’ foliage treatments were designated as factors, for a two-factor crossed analysis of similarities (ANOSIM) with 999 set permutations. ANOSIM is a non-parametric permutation procedure to compare between-groups and within-groups dissimilarities on multivariate data (Clarke and Green, 1988). This procedure calculates an R statistic, wherein *R* = 0, the grouping of treatments is considered random (i.e., there is no interpretable grouping) and *R* = 1, if all replicates within groups are more similar to each other than any replicates between the groups (Klimek et al., 2016). The overall or ‘global’ *R* value was consequently used to express differences as dissimilarity between isolate (BG11, FRh2, J18) and control treatments, and the wounded (W) and non-wounded (N) groups. For the analysis in R (v. 3.2.3), the data was also normalized and converted by Euclidean distance using the Vegan package (v 2.3-5). NMDS plots were then produced for isolate and wounding factorial visualization using Vegan.

## Results

### PCR Sensitivity

**FIGURE 1:**
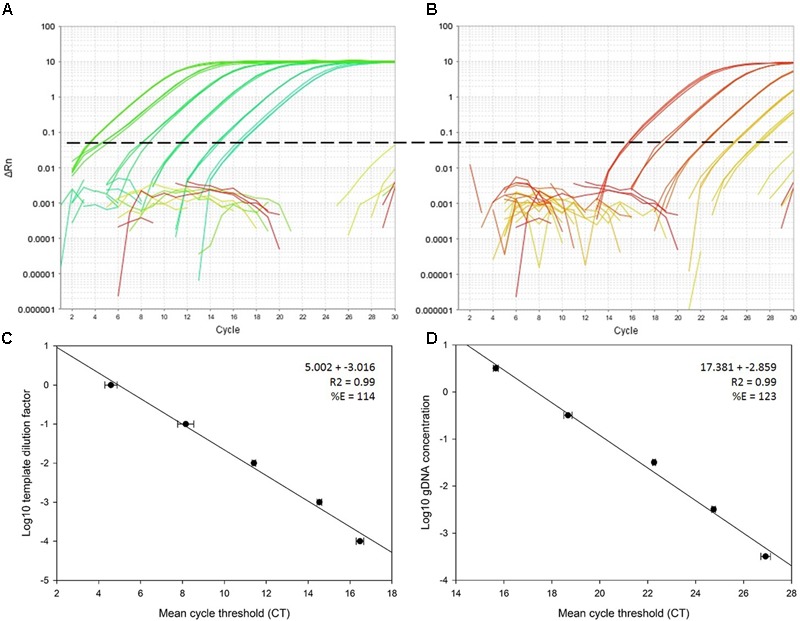
Comparative standard curve analysis of EF4F and EF4R target on a PCR template versus direct amplification of gDNA. Plot **(A)** shows amplification of diluted PCR template produced from a 10-fold dilution series of *Beauveria bassiana* gDNA (isolate J18) spiked in *Zea mays* (maize) gDNA and plot **(B)** shows direct amplification of a 10-fold dilution series of *B. bassiana* gDNA (isolate J18) in H_2_O. Plots **(C)** and **(D)** show the corresponding standard curves produced from the amplification experiments above, with the regression equation and amplification efficiency (%E).

The nested PCR protocol was more sensitive than a single real-time PCR with EF4-4 primers only (**Figure 1**). The standard curve constructed for EF4-4 target amplification on PCR product (EF3-5) template had an amplification fluorescent threshold (FT) at cycle 5.002 on the Y intercept, compared to EF4-4 target amplification of gDNA directly, which had an FT of 17.381. In both standard curves, the PCR amplification efficiency (%E) was over-estimated with 114% for the nested protocol and 123% for the single PCR experiment. Omitting the final dilution (10^-4^), improved the efficiencies to 100% for the nested PCR and 110% for single qPCR using EF4-4 primers.

**FIGURE 2:**
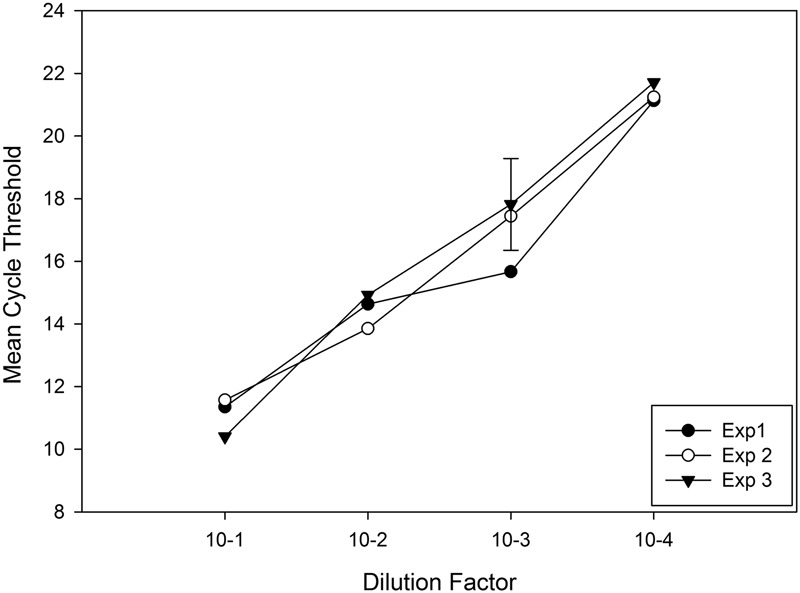
Cycle threshold (CT) means representing three real-time qPCR experiments for elongation factor 1-α, *ef1α*, (primers EF4F and EF4R) amplifying DNA template (diluted 1/1000 μL H_2_O) generated from PCR (primers EF3F and EF5R) of *Beauveria bassiana* mixed into *Zea mays* DNA, prepared prior in a 10-fold dilution series, to determine consistency between PCR experiments and replicates. A 5% least significant difference (LSD) bar compares PCR experiments and replicates for significance.

Statistical analysis of three qPCR amplification experiments performed on template produced from mixed DNA (EF3-5; *B. bassiana* and maize) showed no significant differences in the ANOVA that tested mean cycle threshold (CT) values from each standard curve (*P* = 0.346; 5% LSD = 1.464) (**Figure 2**). The linear equation averaged for three replicated real time PCR experiments (nested protocol) was 12.32 + -2.763x; R2 = 0.99, representing the target amplification range for detection of *B. bassiana* in maize DNA.

### PCR Specificity

**FIGURE 3:**
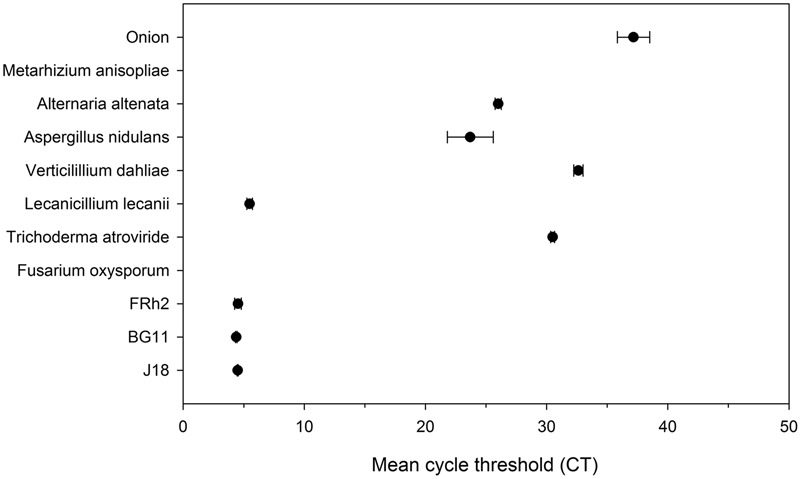
Real time qPCR amplification of multiple fungal species and plant host DNA (onion and maize), versus *Beauveria bassiana* isolates (BG11, FRh2, J18) (*Y* axis) over mean cycle threshold (CT) values (*X* axis) with standard deviation error bars (where possible). Amplification was performed using EF4F and EF4R as nested step 2 PCR on product generated from primers EF3F and EF5R.

Real-time amplification of the PCR product generated by primers EF3-5 using the internal EF4-4 primers demonstrated that the nested protocol was specific for the three *B. bassiana* isolates and for the *L. lecanii* (**Figure 3**) under 22 cycles. Using the nested protocol, amplification of the non-target DNA samples began to occur after 22 cycles in a real time experiment, whereas, the target DNA (i.e., *Beauveria*) was detected prior to 5 cycles.

### *Beauveria* in Rhizosphere Soil and Roots

**FIGURE 4:**
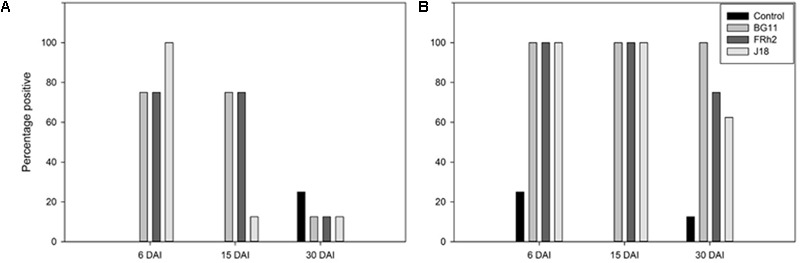
Percentage of *ef1α Beauveria bassiana* detected by PCR in rhizosphere soil **(A)** and on/in the root tissue **(B)** of *Zea mays* (maize) over three sampling events, 6, 15, and 30 days after inoculation (DAI) (*X* axes). The number of positive samples were summed for each *B. bassiana* isolate treatment and control, and then calculated as percent present over samples (*Y* axes).

In soil, a decline in the frequency of detection of *Beauveria ef1α* by PCR was observed by 30 DAI, indicating temporal differences in the inoculum levels. Samples confirmed positive for *B. bassiana* in the rhizosphere by PCR and sequence analysis (data not shown), were summed and then calculated as percent present in samples for each isolate treatment and control, over the three sampling times: 6, 15, and 30 DAI (**Figure 4A**). Detection of *B. bassiana* was more frequent across all treatments in 6 and 15 DAI soil DNA compared to 30 DAI soil DNA. In control soil, *Beauveria* was detected at 30 DAI, but not in 6 and 15 DAI rhizosphere soils.

The detection frequency of *Beauveria ef1α* on/in root DNA by PCR was higher than that observed in the rhizosphere soil samples. Samples confirmed positive in the roots by PCR and nucleic acid sequence analysis were also summed and calculated as percent present, as for the soil data (**Figure 4B**). In 6 and 15 DAI samples, *B. bassiana* was confirmed present in 100% of inoculated root samples. By 30 DAI, only BG11 treated roots were 100% positive, whereas the isolates FRh2 and J18 were less frequently detected.

Although some non-target amplification occurred, nucleotide analysis (visualization in Geneious^®^ Pro 5.6.5 and BLASTn; NCBI) enabled the differentiation of *Beauveria* species (but not isolates) from non-targets (including *L. lecanii*), and the number of fungi from other genera that were amplified using the nested *ef1α* protocol was restricted to three genera approximately (see McKinnon, 2017). The EF4-4 sequence amplicons were generally too short for submission to the NCBI nucleotide database, however, only sequences which exactly matched *B. bassiana* were included in the data analysis.

**FIGURE 5:**
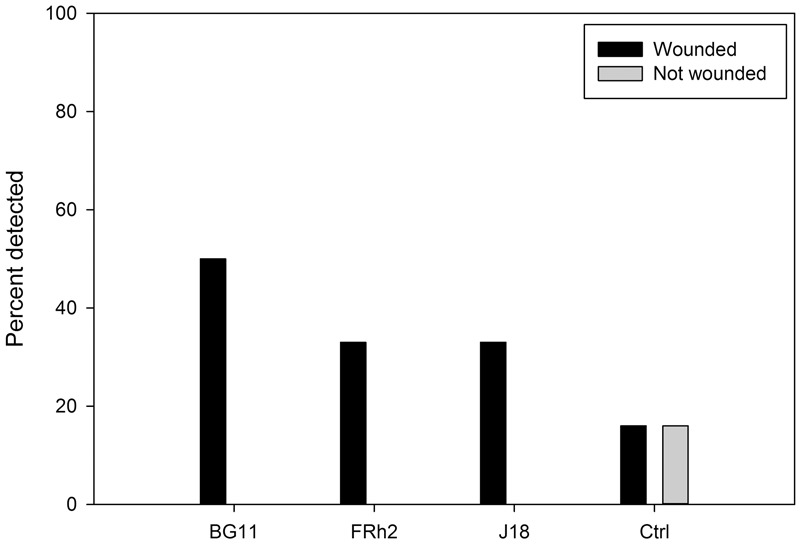
Percentage of *ef1α Beauveria bassiana* detected by PCR in samples from the rhizosphere of *Zea mays* (maize) (*Y* axis) at 30 days after inoculation (DAI), for different isolate treatments versus a no-inoculum control (*X* axis). Compared are the percentage of *B. bassiana* present in soils from plants that were wounded versus those not-wounded (legend).

Despite background levels of *Beauveria* in the control soils at 30 DAI (16.6% presence), when the 30 DAI rhizosphere soil DNA data was analyzed independently in the GLM, there was a statistically significant difference in detection frequency of *Beauveria* from the rhizosphere of wounded (defoliated) plants compared to plants non-wounded (*Z* = 2.332 with *P* = 0.019) (**Figure 5**). The model was supported by the analysis of deviance (*X*^2^; *P* = 0.006). Indeed in all three *B. bassiana* isolate treatments, no *Beauveria* was detected by PCR at 30 DAI in the rhizosphere of plants that were not subjected to defoliation wound-stress. However, there was no significant difference between the isolates *per se* (*P* = 0.54) even though isolate BG11 had a higher frequency (50%) in wounded plants compared to FRh2 and J18 (both at 33%).

### Impacts on the Microbial Community Composition

**FIGURE 6:**
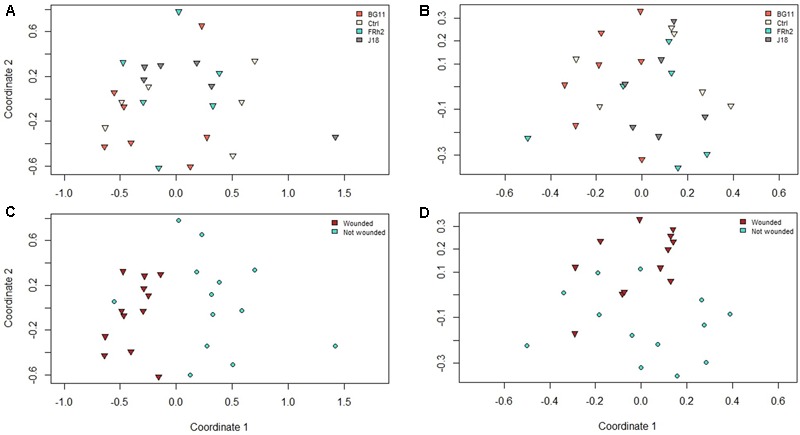
Non-metric multiple dimension scaling (nMDS) plots representing dissimilarities between PCR amplicon profiles from denaturant gradient gel electrophoresis (DGGE) experiments on samples taken from *Zea mays* (maize) rhizosphere soil DNA, at 30 days after inoculation (DAI), with *Beauveria bassiana* isolate treatments indicated (see plots legends) for **(A)** arbuscular mycorrhizal fungi and **(B)** total fungi, and plant wound treatments indicated for **(C)** arbuscular mycorrhizal fungi and **(D)** total fungi.

**FIGURE 7:**
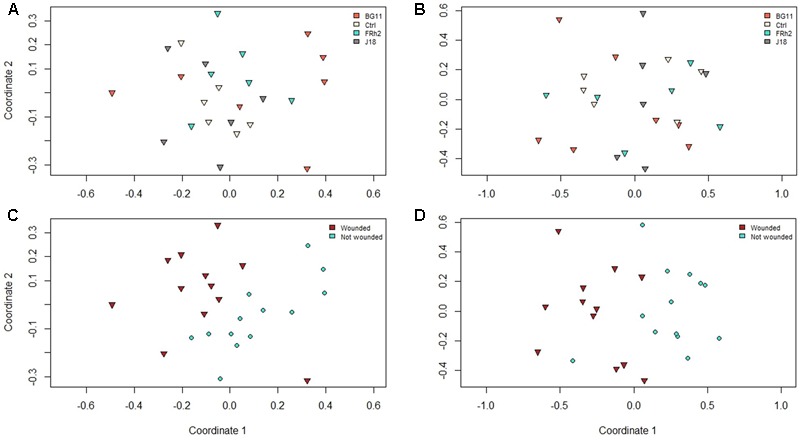
Non-metric multiple dimension scaling (nMDS) plots representing dissimilarities between PCR amplicon profiles from denaturant gradient gel electrophoresis (DGGE) experiments on samples taken from *Zea mays* (maize) rhizosphere soil DNA, at 30 days after inoculation (DAI), with *Beauveria bassiana* isolate treatments indicated (see plots legends) for **(A)** Alphaproteobacteria and **(B)** Betaproteobacteria, and plant wound treatments indicated for **(C)** Alphaproteobacteria and **(D)** Betaproteobacteria.

Visualization of the soil profiles by nMDS (Jaccard similarity) obtained from DGGE indicated no grouping of the microbial communities as a result of the *B. bassiana* isolate inocula treatment factor, however, some grouping of the microbial communities was observed as a result of the wounding treatment (**Figures 6, 7**).

This was supported by the analysis of variance (Adonis) using species richness for all groups (AMF, total fungi, Alphaproteobacteria and Betaproteobacteria), with no significant difference for the isolate treatment (*P* = 0.26) but marginal differences resulting from the wounding treatment as a factor (*P* = 0.07), although this was not significant to the 5% level.

Univariate analysis of each target group demonstrated that species richness may have been slightly higher in the rhizosphere of wounded plants (30 DAI) compared to non-wounded plants for the total fungi group (*P* = 0.003). However, for the Alphaproteobacteria group, mean species richness was higher in the rhizosphere of control plants (mean number of bands/taxa = 29), irrespective of wounding, compared to isolate BG11 treated plants (mean number of bands/taxa = 20; *P* = 0.002), and also higher in the isolate FRh2 treatment (mean number of bands/taxa = 25) compared to BG11 (*P* = 0.04).

### Impacts on the Microbial Community Functional Diversity

**FIGURE 8:**
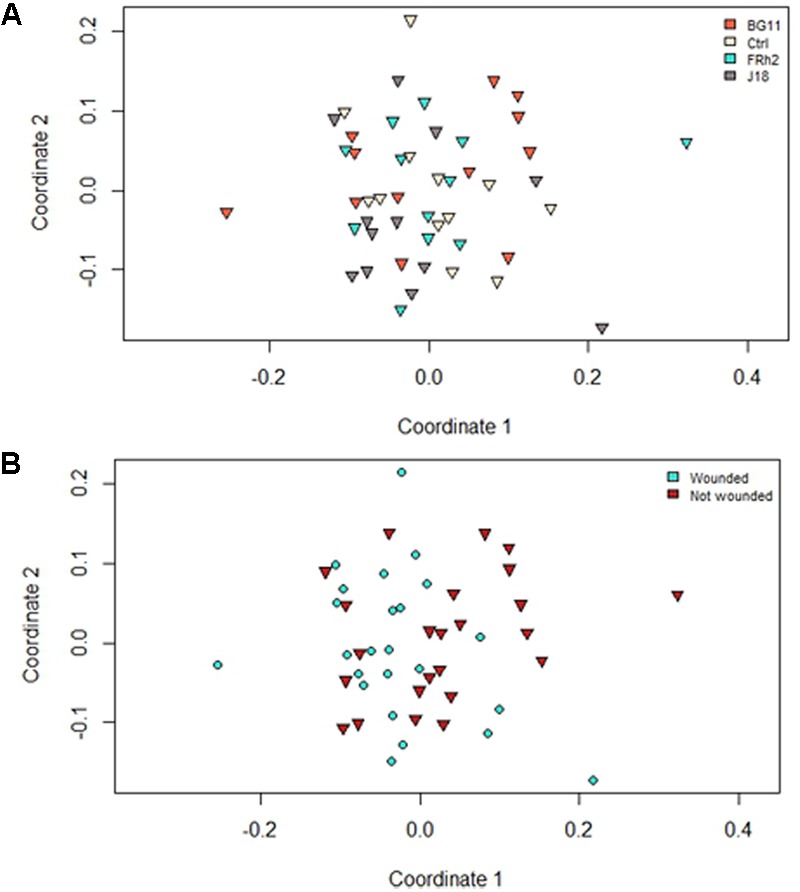
Non-metric multiple dimension scaling plots representing differences between respiration profiles of the microbial communities from rhizosphere soil of *Zea mays* (maize) sampled 30 days after inoculation (DAI) with *Beauveria bassiana*. Data are based on distance matrices obtained from MicroResp^TM^ colorimetric readings. Plots are distinguished by either isolate **(A)** or wound **(B)** treatments as grouping factors.

Functional diversity within the rhizosphere of maize was not affected by presence of the *B. bassiana* inocula. For instance, the two-factor analysis of similarities of MicroResp^TM^ data showed statistical significance to the 5% level (*R* = 0.079, *P* = 0.05) to support no differences between the isolate treatments and control, irrespective of the wounding treatment. However, pairwise comparisons produced subsequently suggested minor differences between BG11 and Ctrl (control) treatments (*R* = 0.196, *P* = 0.01) for the ‘isolate’ factor. All other pairwise comparisons between isolate treatments were not significant. The wounding of foliage (W and N), however, may have resulted in changes in soil carbon utilization in the rhizosphere, as some grouping was observed to suggest marginal differences (*R* = 0.133, *P* < 0.001) (**Figure 8**).

## Discussion

This study compared the impact of different *B. bassiana* isolates on maize to investigate the ecological role of *Beauveria* in association with the plant soil-microbiome. This was accomplished by investigating how inundative inoculation of roots influenced the rhizosphere microbial community structure and function and how, in turn, plants may support root colonization of insect pathogenic fungi in the rhizosphere when subjected to wound stress. Additionally, a sensitive and selective PCR-based detection method was developed for direct detection of multiple *Beauveria* species from either maize or soil DNA. The nested PCR protocol was consequently optimized to minimize non-target amplification while still remaining sensitive enough to amplify less than 0.32 pg/μL, or between 1 and 17 *ef1α* gene copies of *Beauveria*.

As previously mentioned, Landa et al. (2013) developed a PCR/qPCR protocol to monitor *B. bassiana* in opium poppy tissues (see also Quesada-Moraga et al., 2014). Their method also used a two-step nested-PCR approach to target the ITS region that was able to detect as little as 10 femtogram (fg) of *B. bassiana* DNA from the plant leaves. While the ITS primer sets published by Landa et al. (2013) have recently been used successfully with maize leaf tissue for the detection of *B. bassiana* (Tall and Meyling, 2018), the protocol reported in this present study was designed for use with multiple species of *Beauveria*, in order to be able to use, detect and differentiate (by sequence alignment and BLASTn) other species of *Beauveria* (and also *Lecanicillium* and *Isaria*) which may be ubiquitous in rhizosphere soil communities. Because the *ef1α* nested PCR protocol developed can target a broader range of *Beauveria* species as well as a limited number of other entomopathogenic fungi, there is potential to utilize the method to screen plant/rhizosphere material for rapid detection of various novel plant-associated entomopathogens, or to monitor bio-inoculants introduced to plant and soil.

The *ef1α* gene primers and nested PCR protocol developed herein demonstrated a high level of sensitivity and specificity for monitoring *Beauveria* in maize DNA and in non-sterile soil, and was able to amplify multiple *Beauveria* species over 30 days following inoculation while excluding maize and other non-*Beauveria* fungal DNA, as well as onion, tomato and *Arabidopsis* when employed in other studies (McKinnon et al. unpublished). Although studies that have previously used PCR for detection of entomopathogenic fungi from plant material directly are relatively limited, methods for the molecular detection of *Beauveria* from soil have been recently reported (Schwarzenbach et al., 2007; Canfora et al., 2016). These studies used multiple primer sets designed from simple sequence repeat (SSR) markers to be specific to the species targeted, for both qualitative and quantitative detection from soil but not from mixed plant DNA.

Previous literature pertaining to *Beauveria* indicated that these fungi may not be active *per se* in rhizosphere soils, in comparison with species of *Metarhizium* (Bruck, 2010; Kepler et al., 2017). However, in our study *B. bassiana* was more frequently detected in rhizosphere soil when plants were subjected to wound stress, which suggests that these fungi may be functional in the rhizosphere when facilitated by the host plant. As plants are known to antagonize insect herbivores directly by affecting their fecundity via volatile emission, as well as indirectly by recruiting natural enemies of herbivores in the rhizosphere (Dudareva et al., 2006; Howe and Jander, 2008), it is plausible that *B. bassiana* may be recruited and supported by plants within the rhizosphere as a natural enemy of insect herbivores. Although there are preliminary studies that investigate possible mechanisms for plant-derived carbohydrate utilization by *Metarhizium* (Fang and St. Leger, 2010; Pava-Ripoll et al., 2011), as of yet, there are no published studies investigating this for *Beauveria.*

Elliot et al. (2000) first raised the hypothesis that plants may use entomopathogenic fungi as ‘bodyguards.’ Indeed, the results presented here support the theory that recruitment of entomopathogenic fungi by plants may occur as an indirect defensive strategy against herbivory stress. Although the results presented here are preliminary, further studies are certainly warranted to explore the impact that insect herbivory may have on rhizosphere recruitment or retention of entomopathogenic fungi by plants. Our results also support the findings of Keyser et al. (2014), whom tested whether root colonization by *Metarhizium* could be an adaptive strategy in these fungi to increase exposure to plant-associated insects and thus aid their dispersal. Specifically, they tested the pathogenicity of the fungi to *Tenebrio molitor* larvae by exposing the insects to wheat roots that were inoculated prior with *Metarhizium* spp. as a seed treatment. Since the fungi were shown to disperse with roots and retain pathogenicity for up to 4 weeks from inoculation, they asserted that a plant-root association provides a benefit to the fungi by increasing the likelihood of encountering a susceptible insect host. Enhanced rhizosphere colonization of *Beauveria* spp. in plants experiencing insect herbivory may also therefore be an adaptive strategy of these fungi to increase the likelihood of encountering susceptible insect hosts.

These bodyguard and dispersal hypotheses (Elliot et al., 2000; Keyser et al., 2014) were also supported by the results of the DGGE and MicroResp^TM^ assays, in which structural and functional diversity was altered by those plants under distress from the above-ground damage to foliage, as demonstrated by observed differences in species richness (total fungi and Alphaproteobacteria) and the slight differences in carbon utilization profiles (for the wounding treatment only).

Plant systemic resistance or susceptibility may be induced in certain organs and tissues following pathogen or insect attack that result in the activation of signaling pathways to affect distant tissues, and can subsequently also affect below-ground microbes (Doornbos et al., 2011). For example, in pepper plants, herbivory by sap-sucking whiteflies or aphids has been shown to induce the up-regulation of transcription factors that govern both SA- and JA-dependent pathways in leaves and in roots (Lee et al., 2012; Yang et al., 2012). Furthermore, plants can actively recruit non-pathogenic root-associated microbes following attack by insects or pathogens through the modulation of these specific hormone signaling pathways (Rudrappa et al., 2008; Lakshmanan et al., 2012; Lee et al., 2012). An insect-induced belowground plant signal, (E)-β-caryophyllene, was reported to be emitted by maize roots in response to root-feeding by beetle larvae of *Diabrotica virgifera virgifera* in a study by Rasmann et al. (2005). The (E)-β-caryophyllene was shown to strongly attract an entomopathogenic nematode. It is plausible therefore, that induced systemic susceptibility and/or volatile emission in plants resulting from herbivory may facilitate endophytic or rhizosphere colonization from entomopathogenic fungi such as *Beauveria*, albeit indirectly. As a result, the associated insect herbivores may have a higher risk of contracting infection by these insect pathogens.

Following activation of defense pathways, plants can release a suite of volatiles that specifically attract natural enemies of the herbivores (Arimura et al., 2009; War et al., 2012). For example, Lakshmanan et al. (2012) observed higher levels of beneficial rhizobacteria *Bacillus subtilis* in the rhizosphere of *Arabidopsis thaliana* when the plants were subjected to stress from foliar pathogen attack by *Pseudomonas syringae*. Foliar infection by the pathogen induced the expression of a malic acid transporter resulting in an increase of malic acid in the rhizosphere. Furthermore, the biofilm formation in *B. subtilis* on roots actually negated the suppression of microbe-associated molecular patterns (MAMPs), allowing continued defense against the disease. A study conducted on ragwort plants (*Jacobea vulgaris*) showed that both above- and below-ground herbivory altered the composition of the soil fungal community, which was attributed to changes in root exudates (Kostenko et al., 2012). Evidence that insect herbivory can influence root-associated microbes via changes in root exudation has also been reported for maize plants. Root-feeding by western corn rootworm (WCR) larvae was shown to change the composition of the microbial community in the rhizosphere when analyzed by DGGE (Dematheis et al., 2012).

Future studies are therefore warranted to investigate the specific mechanisms that may be involved in maize and in *B. bassiana* to enable persistence in the rhizosphere under stress. For example, studies to determine the precise root exudate or components that enhance rhizosphere retention during herbivory and/or wound stress, would not only be interesting from an evolutionary ecology perspective, but may also support the formulation of these exudates for practical application to enhance rhizosphere colonization by these fungi, to enhance biocontrol outcomes. It would be beneficial to assess the relationship between insect herbivore mortality and/or fecundity for plants subjected to extensive herbivory damage compared to healthy (untouched) plants, in order to elucidate and quantify the biocontrol potential of rhizosphere colonization.

It would also be useful to determine more precisely any microbiome alterations following wounding to foliage and in the presence of the *Beauveria* inocula, by utilizing next generation sequencing technology such as metabarcoding of the rhizosphere samples. Although DGGE proved useful to determine preliminary community changes overall, the question of which taxa may be affected specifically, still requires elucidation. Minor changes in carbon utilization profiles may or may not reflect meaningful differences in microbial diversity, depending upon the specific taxa that are either displaced or enhanced as a result of the plant stress response or inocula treatments. For instance, a recent study by Mahoney-Kurpe (2017) used metabarcoding to ascertain impacts of *Epichloë* endophyte inoculation on the rhizosphere microbiota associated with perennial ryegrass, and found that only one class of fungi, likely the Sodariomycetes (Pezizomycotina; Ascomycota), was negatively affected with respect to abundance in the infected-plant treatment. Overall, this effect was statistically insignificant between infected and non-infected plant treatments, however, the functional role (if any) of the Sodariomycetes in the rhizosphere was not determined.

In this present study, the isolates had no or little influence on the microbial community composition and function in the rhizosphere by 30 DAI (DGGE and MicroResp^TM^ analyses). Given that a significant decline in all detectable *Beauveria* was observed by 30 DAI using PCR, this was not unexpected but may indicate that initial inocula levels did not have a lasting effect. A recent study by Hong et al. (2017) also demonstrated that an application of *M. anisopliae* had a minimal impact on the endogenous microbial diversity of rice plants within the phyllosphere, with only transient changes in bacterial abundance/diversity that may have resulted in some small benefits to the plant. Thus multitrophic interactions are important to consider, in addition to any possible ecosystem impacts that may influence performance of a biocontrol agent applied to agroecosystems (Ownley et al., 2010). However, there is limited information on interactions between entomopathogenic fungi and other plant associated microorganisms apart from pathogens (Ownley et al., 2008; Lozano-Tovar et al., 2017). A recent study by Zitlalpopoca-Hernandez et al. (2017) recovered *B. bassiana* from soil of potted maize plants after 7 weeks. In this instance, the soil (soil:sand) was inoculated with the *Beauveria* using a conidial suspension and mixed through prior to planting. When AMF was dual-inoculated with the *B. bassiana* soil inoculum, the population density of *B. bassiana* in soil was lower than when *Beauveria* was used singularly. This observed reduction in population density may have been a result of competitive exclusion of the plant-derived nutrients by the AMF, suggesting that *Beauveria* soil populations rely on plant roots to some extent for nutrients. However, an earlier study by Gualandi et al. (2014) reported that the inoculation of AMF did not affect *B. bassiana* endophytic colonization in the roots of *Echinacea*, which is more consistent with the results of this present study since *Beauveria* was more frequently detected by PCR analysis in/on roots than in rhizosphere soil directly.

## Conclusion

The most common approach for use of entomopathogenic fungi is to apply inundatively, but the fungi often perform inconsistently in the field. This may be due in part to a lack of understanding of their ecology and biology, in addition to the expectation that they should perform similarly to synthetic pesticides (Roy et al., 2010). Historically, biocontrol isolates of *B. bassiana* have been selected for release in the field based solely on their efficacy in laboratory bioassays, irrespective of their microhabitat preferences and ecological constraints (Bidochka et al., 2001). More recently, evidence has accumulated for the potential to use endophytic entomopathogenic fungi for biocontrol purposes (Vidal and Jaber, 2015) and thus there is increasing importance to understand the ecology and complete life history of these fungi in association with plants. Our study demonstrated that *B. bassiana* may be active in the rhizosphere of maize when under stress from herbivory and this may have implications for enhancing biocontrol outcomes, as well as provide resolution into the life history of function of these fungi apart from insect epidemiology. The questions remaining here for investigation are what microbial species of the microbiome are included or excluded as a result of the presence of these inocula (if any), and more particularly, are insect herbivores more at risk of infection by *Beauveria* as a result of this interaction in the rhizosphere?

## Author Contributions

ACM conducted and authored this research. TG, HR, AM-M, and AH supervised and assisted the research. TG developed the original project. HR led the MicroResp and DDGE analyses. AH assisted with primer design and qPCR protocols and AM-M assisted with maize experimental techniques. WG and JB provided guidance for the statistical analyses. ACM drafted the manuscript and all authors contributed.

## Conflict of Interest Statement

The authors declare that the research was conducted in the absence of any commercial or financial relationships that could be construed as a potential conflict of interest.

## References

[B1] Altschul S. F. Gish W. Miller W. E. Myers W. Lipman D. J. (1990). Basic local alignment search tool. *J. Mol. Biol.* 215 403–410. 10.1016/S0022-2836(05)80360-2 2231712

[B2] Altschul S. F. Madden L. T. Schäffer A. A. Zhang J. Zhang Z. Miller W. (1997). Gapped BLAST and PSI-BLAST: a new generation of protein database search programs. *Nucleic Acids Res.* 25 3389–3402. 10.1093/nar/25.17.3389 9254694 PMC146917

[B3] Arimura G. Matsui K. Takabayashi J. (2009). Chemical and molecular ecology of herbivore-induced plant volatiles: proximate factors and their ultimate functions. *Plant Cell Physiol.* 50 911–923. 10.1093/pcp/pcp030 19246460

[B4] Bacon C. W. White J. F. (2016). Functions, mechanisms and regulation of endophytic and epiphytic microbial communities of plants. *Symbiosis* 68 87–98. 10.1007/s13199-015-0350-2

[B5] Barelli L. Moonjely S. Behie W. S. Bidochka M. J. (2016). Fungi with multifunctional lifestyles: endophytic insect pathogenic fungi. *Plant Mol. Biol.* 90 657–664. 10.1007/s11103-015-0413-z 26644135

[B6] Behie S. W. Padilla-Guerrero I. E. Bidochka M. J. (2013). Nutrient transfer to plants by phylogenetically diverse fungi suggests convergent evolutionary strategies in rhizospheric symbionts. *Commun. Integr. Biol.* 6:e22321. 10.4161/cib.22321 23802036 PMC3689567

[B7] Behie S. W. Zelisko M. P. Bidochka M. J. (2012). Endophytic insect-parasitic fungi translocate nitrogen directly from insects to plants. *Science* 336 1576–1577. 10.1126/science.1222289 22723421

[B8] Bidochka M. J. Kamp A. M. Lavender T. M. Dekoning J. De Croos J. N. (2001). Habitat association in two genetic groups of the insect-pathogenic fungus *Metarhizium anisopliae*: uncovering cryptic species? *Appl. Environ. Microbiol.* 67 1335–1342. 10.1128/AEM.67.3.1335-1342.2001 11229929 PMC92732

[B9] Brookes J. J. (2017). *Endophytes in Maize (Zea mays) in New Zealand.* Lincoln: Lincoln University.

[B10] Brown S. D. Collins R. A. Boyer S. Lefort M.-C. Malumbres-Olarte J. Vink C. J. (2012). Spider: an R package for the analysis of species identity and evolution, with particular reference to DNA barcoding. *Mol. Ecol. Resour.* 12 562–565. 10.1111/j.1755-0998.2011.03108.x 22243808

[B11] Bruck D. J. (2010). Fungal entomopathogens in the rhizosphere. *Biocontrol* 55 103–112. 10.1007/s10526-009-9236-7

[B12] Calbrix R. Laval K. Barray S. (2005). Analysis of the potential functional diversity of the bacterial community in soil: a reproducible procedure using sole-carbon-source utilization profiles. *Eur. J. Soil Biol.* 41 11–20. 10.1016/j.ejsobi.2005.02.004

[B13] Campbell C. D. Chapman S. J. Cameron C. M. Davidson M. S. Potts J. M. (2003). A rapid microtiter plate method to measure carbon dioxide evolved from carbon substrate amendments so as to determine the physiological profiles of soil microbial communities by using whole soil. *Appl. Environ. Microbiol.* 69 3593–3599. 10.1128/aem.69.6.3593-3599.2003 12788767 PMC161481

[B14] Canfora L. Malusà E. Tkaczuk C. Tartanus M. Łabanowska B. H. Pinzari F. (2016). Development of a method for detection and quantification of *B. brongniartii* and *B. bassiana* in soil. *Sci. Rep.* 6:22933. 10.1038/srep22933 26975931 PMC4791642

[B15] Chapman S. J. Campbell D. C. Artz R. R. E. (2007). Assessing CLPPs using MicroResp^TM^. *J. Soils Sediments* 7 406–410. 10.1065/jss2007.10.259

[B16] Clarke K. R. Green R. H. (1988). Statistical design and analysis for a biological effects study. *Mar. Ecol. Prog. Ser.* 46 213–226. 10.3354/meps046213

[B17] Clarke K. R. Gorley R. N. (2015). *PRIMER v7: User Manual/Tutorial* (Plymouth: PRIMER-E) 296.

[B18] Degens B. P. Harris J. A. (1997). Development of a physiological approach to measuring the catabolic diversity of soil microbial communities. *Soil Biol. Biochem.* 29 1309–1320. 10.1016/S0038-0717(97)00076-X

[B19] Dematheis F. Zimmerling U. Flocco C. Kurtz B. Vidal S. Kropf S. (2012). Multitrophic interaction in the rhizosphere of maize: root feeding of western corn rootworm larvae alters the microbial community composition. *PLoS One* 7:e37288. 10.1371/journal.pone.0037288 22629377 PMC3358342

[B20] Doornbos R. F. Geraats P. B. Kuramae E. E. Van Loon L. C. Bakker P. A. (2011). Effects of jasmonic acid, ethylene, and salicylic acid signaling on the rhizosphere bacterial community of *Arabidopsis thaliana*. *Mol. Plant Microbe Interact.* 24 395–407. 10.1094/mpmi-05-10-0115 21171889

[B21] Dudareva N. Negre F. Nagegowda A. D. Orlova I. (2006). Plant volatiles: recent advances and future perspectives. *Crit. Rev. Plant Sci.* 25 417–440. 10.1080/07352680600899973

[B22] Elliot S. L. Sabelis W. M. Janssen A. van der Geest L. P. S. Beerling E. A. Fransen J. M. (2000). Can plants use entomopathogens as bodyguards? *Ecol. Lett.* 3 228–235. 16697914

[B23] Fang W. G. St. Leger R. J. (2010). *Mrt*, a gene unique to fungi, encodes an oligosaccharide transporter and facilitates rhizosphere competency in *Metarhizium robertsii*. *Plant Physiol.* 154 1549–1557. 10.1104/pp.110.163014 20837701 PMC2971628

[B24] Gomes N. C. M. Heuer H. Schonfeld J. Costa R. Mendonca-Hagler L. Smalla K. (2001). Bacterial diversity of the rhizosphere of maize (*Zea mays*) grown in tropical soil studied by temperature gradient gel electrophoresis. *Plant Soil* 232 167–180. 10.1023/a:1010350406708

[B25] Gualandi R. J. Augé R. M. D. Kopsell A. Ownley H. B. Chen F. Toler D. H. (2014). Fungal mutualists enhance growth and phytochemical content in *Echinacea purpurea*. *Symbiosis* 63 111–121. 10.1007/s13199-014-0293-z

[B26] Haney C. H. Wiesmann C. L. Shapiro L. Melnyk A. R. O’Sullivan L. R. Khorasani S. (2017). Rhizosphere-associated *Pseudomonas* induce systemic resistance to herbivores at the cost of susceptibility to bacterial pathogens. *Mol. Ecol.* 27 1833–1847. 10.1111/mec.14400 29087012

[B27] Higgins D. G. Sharp P. M. (1988). CLUSTAL: a package for performing multiple sequence alignment on a microcomputer. *Gene* 73 237–244. 10.1016/0378-1119(88)90330-7 3243435

[B28] Hirsch J. Galidevara S. Strohmeier S. Devi U. K. Reineke A. (2013). Effects on diversity of soil fungal community and fate of an artificially applied *Beauveria bassiana* strain assessed through 454 pyrosequencing. *Microb. Ecol.* 66 608–620. 10.1007/s00248-013-0249-5 23736813

[B29] Hong M. Peng G. Keyhani O. N. Xia Y. (2017). Application of the entomogenous fungus, *Metarhizium anisopliae*, for leafroller (*Cnaphalocrocis medinalis*) control and its effect on rice phyllosphere microbial diversity. *Appl. Microbiol. Biotechnol.* 101 6793–6807. 10.1007/s00253-017-8390-6 28695229

[B30] Howe G. A. Jander G. (2008). Plant immunity to insect herbivores. *Annu. Rev. Plant Biol.* 59 41–66. 10.1146/annurev.arplant.59.032607.092825 18031220

[B31] Hu G. St. Leger R. J. (2002). Field studies using a recombinant mycoinsecticide (*Metarhizium anisopliae*) reveal that it is rhizosphere competent. *Appl. Environ. Microbiol.* 68 6383–6387. 10.1128/aem.68.12.6383-6387.2002 12450863 PMC134390

[B32] Jaronski S. T (2008). “Soil ecology of the entomopathogenic Ascomycetes: a critical examination of what we (think) we know,” in *Use of Entomopathogenic Fungi in Biological Pest Management* ed. Ekesi S. Maniania N. K. (Thiruvananthapuram: Research Signpost) 91–144.

[B33] Kepler R. M. Maul J. E. Rehner S. A. (2017). Managing the plant microbiome for biocontrol fungi: examples from Hypocreales. *Curr. Opin. Microbiol.* 37 48–53. 10.1016/j.mib.2017.03.006 28441534

[B34] Keyser C. A. Thorup-Kristensen K. Meyling N. V. (2014). *Metarhizium* seed treatment mediates fungal dispersal via roots and induces infections in insects. *Fungal Ecol.* 11 122–131. 10.1016/j.funeco.2014.05.005

[B35] Kia S. H. Glynou K. Nau T. Thines M. Piepenbring M. Macia-Vicente J. G. (2017). Influence of phylogenetic conservatism and trait convergence on the interactions between fungal root endophytes and plants. *ISME J.* 11 777–790. 10.1038/ismej.2016.140 27801904 PMC5322293

[B36] Klimek B. Chodak M. Jazwa M. Solak A. Tarasek A. Niklinska M. (2016). The relationship between soil bacteria substrate utilisation patterns and the vegetation structure in temperate forests. *Eur. J. For. Res.* 135 179–189. 10.1007/s10342-015-0929-4

[B37] Koch E. Zink P. Ullrich C. I. Kleespies R. G. (2018). Light microscopic studies on the development of *Beauveria bassiana* and other putative endophytes in leaf tissues. *J. Kulturpflanzen* 70 95–107. 10.1399/JKI.2018.03.02

[B38] Kostenko O. van de Voorde T. F. Mulder P. P. van der Putten W. H. Martijn Bezemer T. (2012). Legacy effects of aboveground-belowground interactions. *Ecol. Lett.* 15 813–821. 10.1111/j.1461-0248.2012.01801.x 22594311

[B39] Lakshmanan V. Kitto S. L. Caplan J. L. Hsueh Y. H. Kearns D. B. Wu Y. S. (2012). Microbe-associated molecular patterns-triggered root responses mediate beneficial rhizobacterial recruitment in *Arabidopsis*. *Plant Physiol.* 160 1642–1661. 10.1104/pp.112.200386 22972705 PMC3486800

[B40] Landa B. B. Lopez-Diaz C. Jimenez-Fernandez D. Montes-Borrego M. Munoz-Ledesma F. J. Ortiz-Urquiza A. (2013). *In-planta* detection and monitorization of endophytic colonization by a *Beauveria bassiana* strain using a new-developed nested and quantitative PCR-based assay and confocal laser scanning microscopy. *J. Invertebr. Pathol.* 114 128–138. 10.1016/j.jip.2013.06.007 23851123

[B41] Lee B. Lee S. Ryu C. M. (2012). Foliar aphid feeding recruits rhizosphere bacteria and primes plant immunity against pathogenic and non-pathogenic bacteria in pepper. *Ann. Bot.* 110 281–290. 10.1093/aob/mcs055 22437662 PMC3394643

[B42] Lee J. Lee S. Young J. P. W. (2008). Improved PCR primers for the detection and identification of arbuscular mycorrhizal fungi. *FEMS Microbiol. Ecol.* 65 339–349. 10.1111/j.1574-6941.2008.00531.x 18631176

[B43] Lozano-Tovar M. D. Garrido-Jurado I. Quesada-Moraga E. Raya-Ortega M. C. Trapero-Casas A. (2017). *Metarhizium brunneum* and *Beauveria bassiana* release secondary metabolites with antagonistic activity against *Verticillium dahliae* and *Phytophthora megasperma* olive pathogens. *Crop Prot.* 100 186–195. 10.1016/j.cropro.2017.06.026

[B44] Lugtenberg B. J. Chin-A-Woeng T. F. Bloemberg G. V. (2002). Microbe-plant interactions: principles and mechanisms. *Antonie Van Leeuwenhoek* 81 373–383. 10.1023/a:1020596903142 12448736

[B45] Mahoney-Kurpe S. (2017). *Metabarcoding of the Rhizosphere Microbiome of Perennial Ryegrass in Response to Epichloë festucae var. lolii Infection.* Masters thesis, Massey University Palmerston North.

[B46] Mayerhofer J. Eckard S. Hartmann M. Grabenweger G. Widmer F. Leuchtmann A. (2017). Assessing effects of the entomopathogenic fungus *Metarhizium brunneum* on soil microbial communities in *Agriotes* spp. biological pest control. *FEMS Microbiol. Ecol.* 93:fix117. 10.1093/femsec/fix117 28961941 PMC5812499

[B47] McKinnon A. C. (2017). *Interactions Between Isolates of the Fungus Beauveria bassiana and Zea mays.* Doctoral thesis, Lincoln University Christchurch.

[B48] McKinnon A. C. Saari S. Moran-Diez M. E. Meyling N. V. Raad M. Glare T. R. (2017). *Beauveria bassiana* as an endophyte: a critical review on associated methodology and biocontrol potential. *BioControl* 62 1–17. 10.1007/s10526-016-9769-5

[B49] Meyling N. V. Eilenberg J. (2007). Ecology of the entomopathogenic fungi *Beauveria bassiana* and *Metarhizium anisopliae* in temperate agroecosystems: potential for conservation biological control. *Biol. Control* 43 145–155. 10.1016/j.biocontro1.2007.07.007

[B50] Muhling M. Woolven-Allen J. Murrell J. C. Joint I. (2008). Improved group-specific PCR primers for denaturing gradient gel electrophoresis analysis of the genetic diversity of complex microbial communities. *ISME J.* 2 379–392. 10.1038/ismej.2007.97 18340335

[B51] Muyzer G. Dewaal C. E. Uitterlinden A. G. (1993). Profiling of complex microbial-populations by denaturing gradient gel-electrophoresis analysis of polymerase chain reaction-amplified genes-coding for 16s ribosomal-RNA. *Appl. Environ. Microbiol.* 59 695–700. 7683183 10.1128/aem.59.3.695-700.1993 PMC202176

[B52] Ownley B. H. Griffin M. R. Klingeman W. E. Gwinn K. D. Moulton J. K. Pereira R. M. (2008). *Beauveria bassiana*: endophytic colonization and plant disease control. *J. Invertebr. Pathol.* 98 267–270. 10.1016/j.jip.2008.01.010 18442830

[B53] Ownley B. H. Gwinn K. D. Vega F. E. (2010). Endophytic fungal entomopathogens with activity against plant pathogens: ecology and evolution. *Biocontrol* 55 113–128. 10.1007/s10526-009-9241-x

[B54] Pangesti N. Pineda A. Pieterse C. M. Dicke M. J. van Loon J. J. A. (2013). Two-way plant-mediated interactions between root-associated microbes and insects: from ecology to mechanisms. *Front. Plant Sci.* 4:414. 10.3389/fpls.2013.00414 24167508 PMC3805956

[B55] Pava-Ripoll M. Angelini C. Fang W. Wang S. Posada F. J. St Leger R. (2011). The rhizosphere-competent entomopathogen *Metarhizium anisopliae* expresses a specific subset of genes in plant root exudate. *Microbiology* 157 47–55. 10.1099/mic.0.042200-0 20947574

[B56] Pieterse C. M. Van der Does D. Zamioudis C. Leon-Reyes A. Van Wees S. C. (2012). Hormonal modulation of plant immunity. *Annu. Rev. Cell Dev. Biol.* 28 489–521. 10.1146/annurev-cellbio-092910-154055 22559264

[B57] Quesada-Moraga E. Lopez-Diaz C. Landa B. B. (2014). The hidden habit of the entomopathogenic fungus *Beauveria bassiana*: first demonstration of vertical plant transmission. *PLoS One* 9:e89278. 10.1371/journal.pone.0089278 24551242 PMC3925241

[B58] Rasmann S. Köllner T. G. Degenhardt J. Hiltpold I. Toepfer S. Kuhlmann U. (2005). Recruitment of entomopathogenic nematodes by insect-damaged maize roots. *Nature* 434 732–737. 10.1038/nature03451 15815622

[B59] Reynolds H. L. Packer A. Bever J. D. Clay K. (2003). Grassroots ecology: plant–microbe–soil interactions as drivers of plant community structure and dynamics. *Ecology* 84 2281–2291. 10.1890/02-0298 12290172

[B60] Roy H. E. Brodie E. L. Chandler D. Goettel S. M. Pell K. J. Wajnberg E. (2010). Deep space and hidden depths: understanding the evolution and ecology of fungal entomopathogens. *Biocontrol* 55 1–6. 10.1007/s10526-009-9244-7

[B61] Rudrappa T. Czymmek J. K. Pare W. P. Bais H. P. (2008). Root-secreted malic acid recruits beneficial soil bacteria. *Plant Physiol.* 148 1547–1556. 10.1104/pp.108.127613 18820082 PMC2577262

[B62] Schulz B. Guske S. Dammann U. Boyle C. (1998). Endophyte-host interactions. II. Defining symbiosis of the endophyte-host interaction. *Symbiosis* 25 213–227.

[B63] Schwarzenbach K. Widmer F. Enkerli J. (2007). Cultivation-independent analysis of fungal genotypes in soil by using simple sequence repeat markers. *Appl. Environ. Microbiol.* 73 6519–6525. 10.1128/aem.01405-07 17720832 PMC2075065

[B64] Simon L. Lalonde M. Bruns T. D. (1992). Specific amplification of 18s fungal ribosomal genes from vesicular-arbuscular endomycorrhizal fungi colonizing roots. *Appl. Environ. Microbiol.* 58 291–295. 1339260 10.1128/aem.58.1.291-295.1992 PMC195206

[B65] Tall S. Meyling V. (2018). Probiotics for plants? Growth promotion by the entomopathogenic fungus *Beauveria bassiana* depends on nutrient availability. *Microb. Ecol.* 1–7. 10.1007/s00248-018-1180-6 [Epub ahead of print]. 29594431

[B66] Ullrich C. I. Koch E. Matecki C. Schäfer J. Burkl T. Rabenstein F. (2017). Detection and growth of endophytic entomopathogenic fungi in dicot crop plants. *J. Kulturpflanzen* 69 291–302. 10.1399/JfK.2017.09.02

[B67] Vainio E. J. Hantula J. (2000). Direct analysis of wood-inhabiting fungi using denaturing gradient gel electrophoresis of amplified ribosomal DNA. *Mycol. Res.* 104 927–936. 10.1017/s0953756200002471

[B68] Vargas W. A. Mandawe C. J. Kenerley C. M. (2009). Plant-derived sucrose is a key element in the symbiotic association between *Trichoderma virens* and maize plants. *Plant Physiol.* 151 792–808. 10.1104/pp.109.141291 19675155 PMC2754623

[B69] Vidal S. Jaber L. R. (2015). Entomopathogenic fungi as endophytes: plant-endophyte-herbivore interactions and prospects for use in biological control. *Curr. Sci.* 109 46–54.

[B70] War A. R. Paulraj G. M. Ahmad T. Buhroo A. A. Hussain B. Ignacimuthu S. (2012). Mechanisms of plant defense against insect herbivores. *Plant Signal. Behav.* 7 1306–1320. 10.4161/psb.21663 22895106 PMC3493419

[B71] Wicaksono W. A. Jones E. E. Monk J. Ridgway H. J. (2016). The bacterial signature of *Leptospermum scoparium* (Manuka) reveals core and accessory communities with bioactive properties. *PLoS One* 11:e0163717. 10.1371/journal.pone.0163717 27676607 PMC5038978

[B72] Yang D. L. Yao J. Mei C. S. Tong X. H. Zeng L. J. Li Q. (2012). Plant hormone jasmonate prioritizes defense over growth by interfering with gibberellin signaling cascade. *Proc. Natl. Acad. Sci. U.S.A.* 109 E1192–E1200. 10.1073/pnas.1201616109 22529386 PMC3358897

[B73] Zhang Z. Schwartz S. Wagner L. Miller W. (2000). A greedy algorithm for aligning DNA sequences. *J. Comput. Biol.* 7 203–214. 10.1089/10665270050081478 10890397

[B74] Zitlalpopoca-Hernandez G. Najera-Rincon M. B. del-Val E. K. Alarcon A. Jackson T. Larsen J. (2017). Multitrophic interactions between maize mycorrhizas, the root feeding insect *Phyllophaga vetula* and the entomopathogenic fungus *Beauveria bassiana*. *Appl. Soil Ecol.* 115 38–43. 10.1016/j.apsoil.2017.03.014

